# Understanding the Antecedents of Knowledge Sharing Behavior From the Theory of Planned Behavior Model: Cross-Cultural Comparisons Between Mainland China and Malaysia

**DOI:** 10.3389/fpsyg.2021.772764

**Published:** 2021-11-12

**Authors:** Min Yang, Ping Xu

**Affiliations:** ^1^School of Business Administration, Fujian Jiangxia University, Fuzhou, China; ^2^School of Teacher Education, Shanwei Institute of Technology, Shanwei, China

**Keywords:** self-efficacy, job security, market orientation, knowledge sharing intention, knowledge sharing behavior, theory of planned behavior

## Abstract

Affected by coronavirus disease (COVID-19), in addition to keeping away the impact of the pandemic on their business practices, many enterprises have proposed relevant measures to protect their employees’ job safety and security. Especially for enterprises with high dependence on knowledge resources, employees’ innovation and knowledge sharing play a vital role. In the context of global economic austerity, how to put forward the corresponding plan of knowledge sharing intention to improve the knowledge sharing behavior of employees for enterprises is worth discussing. Mainland China and Malaysia have different quarantine policies and similar industrial structures. This study examines the awareness of Mainland China vs. Malaysian employees, and evaluates the relationship among self-efficacy, job security, market orientation, knowledge sharing intention, and knowledge sharing behavior from the theory of planned behavior. In this study, a total of 627 Mainland China and 434 Malaysian participants were collected to compare both groups in the development of employees’ knowledge sharing behavior. In this study, a variance-based partial least squares structural equation modeling (PLS-SEM) was performed to test the proposed hypothesis and conduct comparative analysis. The results in both the samples show that self-efficacy, job security, and market orientation have positive and significant effects on knowledge sharing intention; self-efficacy has positive and significant effects on job security; knowledge sharing intention has positive and significant effects on knowledge sharing behavior. Moreover, there are several significant differences between Mainland China and Malaysia in the examinations of path comparisons.

## Introduction

From the perspective of the organizational knowledge base (Grant, 1991, 1996; Spender, 1996; Teece, 2000), knowledge is the foundation for an organizational competitive advantage, which eventually becomes the most important driving factor of organizational value creation (Fang et al., 2010; Lin et al., 2015). However, knowledge essentially exists in individuals (Nonaka and Konno, 1998). Therefore, knowledge transfer among individuals and across organizational boundaries and the storage in and access to knowledge bases have become the rules and practices within organizations, which depend on employees’ knowledge sharing behavior (Hassan et al., 2016). Such knowledge sharing behavior has long been regarded as the most important link between knowledge management factors (Lee and Ahn, 2007; Foss et al., 2010; Ohiorenoya and Obadan, 2014; Wu et al., 2015), and knowledge sharing is viewed as an indicator of knowledge management and organizational learning effectiveness (Bock et al., 2005).

Knowledge sharing is considered to be the key to an organization’s success. Decker et al. (2009) summarized the reasons as follows: (1) demographic statistics: older workers, or employees with some expertise, will take important organizational knowledge away with them when they retire or leave the organization (Leonard and Kiron, 2002; Olstein et al., 2005); (2) technical factors: with the increase in technical complexity, knowledge sharing will become more important for collective learning, which contributes to the design and improvement of modern operating systems (Lynn et al., 1999; Cavusgil et al., 2003; Kanaan et al., 2013); (3) constant fierce competition in the technical environment: there is a need for effective knowledge sharing between projects to reduce the time spent on research and development as well as the time to market (Yeung et al., 1999); (4) global operations: organizational knowledge sharing is necessary to transfer effective problem solutions or experience across global operating units (Paik and Choi, 2005); and (5) empirical studies have suggested the importance of knowledge and knowledge sharing and transfer in establishing and maintaining a competitive advantage in a knowledge-driven society (Davenport et al., 1998; Argote and Ingram, 2000; Goh, 2002; van Aalst and van der Mast, 2002; Ahn and Chang, 2004).

Nevertheless, extensive knowledge sharing within an organization seems to be an exception rather than an inevitable phenomenon (Teh and Yong, 2011; Wu, 2013), and it is an intrinsic tendency of human nature to strive to accumulate knowledge and watch it carefully (Davenport and Prusak, 1998). In particular, some organizations actively or unconsciously place limits on knowledge sharing, and the reason mainly lies in the threat of industrial espionage and the concerns about employees neglecting their main work tasks or experiencing an excessive workload (Constant et al., 1994). In regard to reward systems in organizations, excessive rewards for individual performance may work to the disadvantage of knowledge sharing among employees (Huber, 2001). While the importance of knowledge sharing is becoming increasingly prominent, the key issue is to determine which factors affect or hinder knowledge sharing among staff to enable organizations to design and conduct proper management practices to facilitate and encourage knowledge sharing behavior.

General behavior theory explores the influences and relationships among knowledge sharing intention and antecedent factors, as well as the behavior of knowledge sharing, the theory of planned behavior (TPB) being the most widely adopted by researchers (e.g., Bock et al., 2005; Tohidinia and Mosakhani, 2010; Chen, 2011). The TPB has been used extensively in the past to investigate the general behavior of individuals by researchers such as Moons and De Pelsmacker (2015), who explored electric automobiles, Zemore and Ajzen (2014), who investigated medical abuse, Chien et al. (2014), who explored teachers’ behavior in adopting a student scoring mechanism based on information technology in the classroom, Chen (2013), who conducted an exploration of consumers’ network behavior, and Cheon et al. (2012), who discussed the intention among college students to engage in mobile learning. Furthermore, Özer and Yilmaz (2011) discussed the behavior of accounting personnel adopting new information technology, Kim (2010) studied the adoption of mobile data services, Tsai (2010) explored tourists’ consumption behavior, Picazo-Vela et al. (2010) studied the behavior of adopting online reviews by online stores, middlemen, and customers, and Fu (2009) investigated smoking behavior. There may be small differences in the findings from previous studies, but the results are generally quite consistent, all of them verifying the TPB, which offers a good explanation for and prediction of consumer behavior or the specific behavior of ordinary individuals.

As regards the antecedents of the three main variables of the TPB (attitude, subjective norms, and perceived behavioral control), the perspective of social capital theory has mostly been adopted. For instance, in the study by Fauzi et al. (2018), the antecedents were dissected from the perspective of social capital theory; in addition, Akhavan et al. (2015) decomposed the TPB model and, based on it, combined social capital theory and social exchange theory to extract the antecedent factors affecting “attitude.” Notwithstanding the good explanations and predictions also provided by other models, on the whole, the TPB model has been shown to provide the most complete and in-depth insight into intentions and behaviors and to formulate strategies and implement practices to improve behavioral performance, and producing managerial implications and benefits.

In addition to the differences caused by the epidemic, the cross-cultural perspective can be seen as an important moderator that upholds individual feelings and independence (Rehg et al., 2012; Meyers et al., 2019; Lee et al., 2021; Zhao et al., 2021). As the boundaries and differences between cultures become less pronounced in a global world, proposals to guide such employee knowledge sharing behavior are becoming more widely applicable. There are differences in the development of employees’ knowledge sharing in the context of different countries, even in Asian regions (Shogren et al., 2018; Al-Jubari et al., 2019). Hence, employees from Mainland China and Malaysia were used as samples for a cross-cultural comparative analysis in this study to explore the regional differences in working activities caused by health crises and cultures (Hansen et al., 2012; Rehg et al., 2012; Meyers et al., 2019). Therefore, this study focused on determining employees’ perceptions of self-efficacy, job security, market orientation, knowledge sharing intention, and knowledge sharing behavior.

## Literature Review and Hypotheses Development

### Theory of Planned Behavior

The theory of planned behavior (Ajzen, 1985, 1991) is an extension of the theory of reasoned action (Ajzen and Fishbein, 1980), which is regarded as an important theory for explaining and predicting behavior. Many empirical studies have confirmed that this theory provides support for various behavior types. In the TPB, intention, which is conceptualized as the motivational factor influencing a behavior, is considered to be the antecedent influencing autonomous behavior, while attitude and subjective norms are believed to influence behavior through their influence on intention. In Ajzen (1985, 1991) theory, attitude refers to an individual’s favorable or unfavorable evaluation of a certain behavior. Attitudes toward a behavior and behavioral beliefs describe the subjective probability of a particular outcome that may be produced by the behavior, and evaluations describe the implicit value or reward that an individual believes the outcome may bring. According to the TPB, attitude is an important factor that determines some intentional behaviors, and there is an important link between attitude and behavior. When individuals hold a strong positive attitude toward a certain behavior, their intention to perform the behavior becomes relatively strong (Hartwick and Barki, 1994).

Subjective norm refers to the perceived social pressure (Ajzen, 1991) that often drives individuals to perform a behavior to comply with the standard predicted by the norm. Many research findings have also indicated that subjective norms influence behavioral intentions positively (Bock et al., 2005). Ajzen (1985) believed that individual subjective norms are directly or indirectly influenced by others. For instance, Mathieson (1991) and Hartwick and Barki (1994) found that, when the subjective norms of personal computer (PC) users become stronger, users show stronger intentions to use a PC system. Huber (2001) also argued that individuals’ behaviors are usually influenced or regulated by organizational norms. The rules of a group or organization are often transformed into decision criteria for value. For instance, as knowledge sharing is conducive to the development of professional capabilities, it contributes to the creation of greater value for the organization and other members. Thus, it is usually desirable for organizations to share more new knowledge or expertise. In this study, job security was adopted as an important factor for discussing attitude. In terms of perceived behavioral control, self-efficacy was taken as the influencing factor, despite some researchers having included it in “management support” as an antecedent of subjective norms (Iqbal et al., 2011; Fauzi et al., 2018). Other scholars have asserted that the organizational culture exerts a strong influence (Chen, 2011; Akhavan et al., 2015). Thus, to explore knowledge sharing behavior, this study took market orientation as an important normative factor influencing employees’ knowledge sharing intention and knowledge sharing behavior.

### Knowledge Sharing Intention and Behavior

Ajzen (1991) suggested that the intention behind a particular behavior is a motivational factor, which refers to the amount of effort that an individual is willing to put into performing it. As in the TPB model proposed by Ajzen (1985, 1988), the behavioral intention is assumed to exert a positive effect on the actual behavior. Previous studies have also stated that there is indeed a strong causal relationship between behavioral intention and actual behavior in a wide range of different categories of behaviors (Davis, 1989; Mathieson, 1991; Hartwick and Barki, 1994). The research findings from the meta-analysis of 87 studies conducted by Sheppard and Sherman (1998) showed a mean correlation of 0.53 between intention and behavior. Besides, Lin and Lee (2004); Tohidinia and Mosakhani (2010), and Chen (2011), all applied the theoretical model of the TPB to explore the behavior of knowledge sharing and confirmed the direct influence exerted on it by the intention to engage in knowledge sharing. Thereby, the following hypothesis was proposed for this study:

H1: *Knowledge sharing intention has a positive and significant impact on employees’ knowledge sharing behavior.*

### Job Security

In terms of management factors, Ismail et al. (2012) summarized several factors affecting job security, including resources, management, individuals, rewards, and relationships, and classified safety responsibility as leaders’ responsibility and supervision as a management factor. However, in practice, employees’ personal safety cannot be entirely dependent on managers. Thus, it is considered in this study that employees’ personal sense of safety and responsibility exerts a more direct influence on safety behavior and safety consequences. The sense of safety and responsibility refers to employees’ sense of obligation to ensure safety and their willingness to contribute (Gill and Shergill, 2004), that is, individuals’ willingness to dedicate themselves to establishing organizational safety and assume responsibility for it beyond their own roles (Curcuruto et al., 2016). Thus, the sense of safety and responsibility provides a strong sequence of thought at work. The higher the perceived sense of safety and responsibility is, the more the employees will pay attention to their role in the organization and strive to achieve the goal of organizational safety, for instance, reducing accidents, avoiding damage, or achieving the goal of safety improvement (Curcuruto et al., 2016).

It has been found in previous studies that organizational commitment, organizational identity, and psychological ownership are positively correlated with organizational obedience behavior (Podsakoff et al., 2000; Van Dyne and Pierce, 2004). Based on the social impact theory, the process of influence is provided with a similar psychological mechanism as the sense of job security. When organizational members believe in and value the prioritization of job security, they will produce psychological identity and internalize it into their professional roles; thus, they will have a strong sense of reciprocity in organizational knowledge sharing. Although the study by Smith et al. (2016) demonstrated the positive relationship between the safety climate and the behavior of safety obedience, it did not touch on the relationship between employees’ internal psychological mechanism and their intention to share knowledge. In an organization with a high level of job security, signals of safety priority are transmitted through social interactions between administrators and members, and these signals also indicate respect for and care of each other within the organization. Thus, greater organizational commitment and identity among members mean a stronger sense of responsibility and obligation of knowledge sharing and a high degree of knowledge sharing intention in turn (Hofmann and Morgeson, 1999; Neal and Griffin, 2006). Therefore, the following hypothesis was proposed in this study:

H2: *Employees’ job security has a positive and significant impact on employees’ knowledge sharing intention.*

### Self-Efficacy

According to self-efficacy scholars, both environmental factors and cognitive factors in a certain context, particularly those beliefs leading to success and behavior, will influence individuals’ behavioral outcomes (Brown et al., 2011; Chin and Rasdi, 2014; Chang and Edwards, 2015; Liguori et al., 2019; Zhao et al., 2021). They referred to these beliefs as “self-efficacy,” which is a significant cognitive variable in individual factors when accounting for individuals’ behaviors (Caesens and Stinglhamber, 2014) and interaction with their surroundings (Lent et al., 2011; Duffy et al., 2014; Chang and Edwards, 2015; Jemini-Gashi et al., 2019). It can also be regarded as the foundation for the motivation of human behaviors (Cordova et al., 2014), mental health, and individual accomplishment (Lent et al., 2011; Liguori et al., 2019). The field of human resources takes a wide application of self-efficacy to probe into employees’ psychological factors in dynamic situations and the impact on task accomplishment and employees’ occupational development (Brown et al., 2011; Caesens and Stinglhamber, 2014; Duffy et al., 2014; Jemini-Gashi et al., 2019; Lee et al., 2021).

The literature related to organizational behavior has shown that, when an individual perceives that the other members of a group view a particular task (such as innovation) as an important goal, the individual will tend to follow the group members and urge himself or herself to achieve the goal (Brown et al., 2011; Kim and Lee, 2013; Lamm et al., 2015). During the process of socialization, proficiency in work and reference objects for learning and inner self-motivation can both reduce the uncertainty caused by new surroundings and effectively stimulate self-efficacy (Caesens and Stinglhamber, 2014; Chin and Rasdi, 2014). Scholars have stated that employees with high confidence in their ability to complete particular tasks are more likely to share valuable knowledge with others (Shao et al., 2015), as they believe that their knowledge contributes to problem solving and the improvement of work efficiency (Bysted, 2013). In the context of innovation, despite service staff being able to choose the level of innovation to adopt on the basis of personal considerations (Bysted, 2013), knowledge sharing intention is necessary to obtain the corresponding knowledge attributes during innovation at any level. When service staff with high self-efficacy perceive that the innovation goal deviates from the common innovation goal of the group (Brown et al., 2011), they will immediately synchronize the innovation goal *via* the knowledge acquired through knowledge sharing intention (Kim and Lee, 2013). With the passing of time and the accumulation of task experience, service staff will turn knowledge sharing intention into a habit of innovative behavior adjustment. Thus, when service staff have higher self-efficacy in service supply, they will regard knowledge sharing as an important task when providing services (Brown et al., 2011; Caesens and Stinglhamber, 2014). In summary, the study inferred the following hypothesis:

*H3: Self-efficacy has a positive and significant impact on employees’ knowledge sharing intention.*

Caesens and Stinglhamber (2014) defined self-efficacy as a belief in one’s individual ability to perform in a certain field. Chang and Edwards (2015) emphasized that it is not the skill that one owns in performing a certain behavior, but the degree of confidence in using the skill to accomplish a task that matters. Rigotti et al. (2008) believed that self-efficacy refers to individuals’ cognition of personal skills and confidence in their ability to accomplish the task in the context of achievement. Individuals with high self-efficacy will have higher expectations of success and persistence as well as a greater perception of job security, while individuals with low self-efficacy will experience negativity, anxiety, a lack of effort, and low achievements (Caesens and Stinglhamber, 2014; Zhao et al., 2021). Many studies have also provided support for the influence of self-efficacy on perceived behavioral control (Shao et al., 2015), as individuals with high levels of confidence are generally more willing to resolve the difficulties that they may encounter while accomplishing a task (e.g., Liu and Huang, 2015). Therefore, the following hypothesis was proposed in this study:

*H4: Self-efficacy has a positive and significant impact on employees’ job security.*

### Market Orientation

The scholars have suggested diversified interpretations of “market orientation.” It is regarded not only as a series of specific information-processing behaviors and activities (Kohli and Jaworski, 1990) but also as a kind of resource (Hunt and Morgan, 1995) or an organizational culture (Narver and Slater, 1990; Deshpandé et al., 1993). Nonetheless, in empirical studies, the scholars have still mostly adopted the definition proposed by Kohli and Jaworski (1990) and Narver and Slater (1990) as the basis for discussing organizational performance or industrial development. In particular, Narver and Slater (1990) defined “market orientation” as an organizational culture that can create value for customers in an efficient and effective way, thus establishing excellent performance for enterprises. Despite the fact that some opportunities are discovered by accident, firms with a history of successful innovation usually collect and evaluate market information to support opportunity recognition. The firms emphasize the spirit of the enterprise culture, which provides a good environment to promote the constant engagement in exploration and experimental activities to acquire new knowledge (Slater and Narver, 1995). Slater and Narver (1995) further stated that successful innovation arises from entrepreneurs’ ability to identify the gap between the market demand and the available market and to fill this gap effectively. Similarly, the scholars have argued that a highly market-oriented culture is more proactive in information acquisition and utilization and will enhance information acquisition and utilization in innovative, predictable, and risk-taking ways to improve firm performance (Keh et al., 2007).

A market-oriented culture provides employees with active access to external information and knowledge. The employees will not be restricted by the environment but will actively influence their surroundings or take actions in response to environmental changes until significant information and knowledge are produced (Parker et al., 2006). The employees will face a higher degree of collection and spread of market information, they will be more active in applying the information, and they will know that people with more information tend to be positive about challenges and actively transform certain adverse situations into a favorable work context in response to demands at work, reducing the negative effects of the workload on them. In other words, an organization with a highly market-oriented culture enables employees to change the situation to a more favorable environment, thus leading to a higher level of job security and good job performance (Seibert et al., 2001; Bindl et al., 2019). In summary, the study inferred the following hypothesis:

*H5: Market orientation has a positive and significant impact on employees’ job security.*

The organizational culture is generally viewed as an important driving factor for knowledge sharing (Orlikowski, 1993; Constant et al., 1996; Huber, 2001), and Mirzaee and Ghaffari (2018) gave the following explanation in particular: sharing behavior is transformed from accumulating and storing knowledge to gain power into rewarding knowledge sharing to improve power. To achieve this, we must try to form an organizational culture that contributes to establishing long-term and trusting relationships. A market-oriented culture will enable organizational members to collect and spread market knowledge and to trust that all the knowledge/information transferred to them is the best, and the transmitters of this knowledge/information can trust that the transferred knowledge/information will be utilized in a proper manner. Organizational members who are situated in the context of a market-oriented culture are more likely than those who are not to collect and share new and innovative ideas and knowledge with others (Kim and Lee, 1995).

*H6: Market orientation has a positive and significant impact on employees’ knowledge sharing intention.*

**FIGURE 1:**
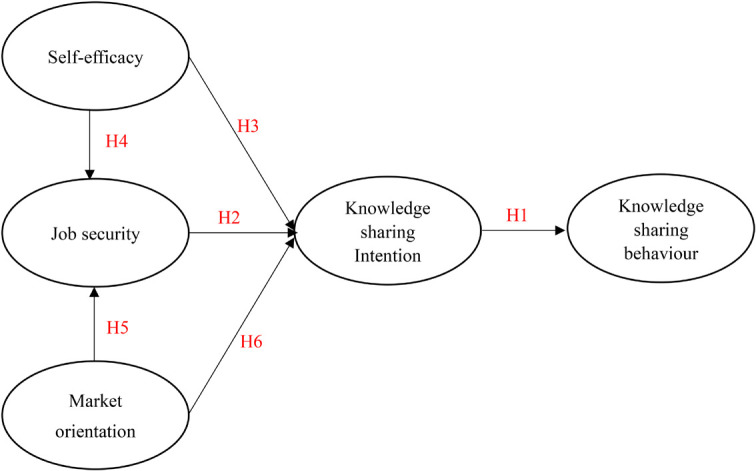
Research framework.

Based on the above hypotheses, this study proposes the following research framework (Figure 1).

## Methodology

### Sampling

The purpose of this research was to explore the employees’ knowledge sharing behavior and to analyze the impacts of the TPB antecedents. The research sample in this study comprised employees who were identified through purposive sampling. A structural model was constructed to explore the correlations among self-efficacy, job security, market orientation, knowledge sharing intention, and knowledge sharing behavior. The sample was obtained from companies in Mainland China and Malaysia. To ensure the representativeness of the sample and reduce the influence that bias derived from the industrial category and job characteristics can bring to the research findings, we distributed copies of the questionnaire to the participants in the information technology industry. As there are large geographical differences and scattered industrial distribution in Mainland China and Malaysia, to obtain more accurate sampling objects, areas in Guangdong were selected as partial sampling areas in Mainland China as well as cities along the West Asian coast in Malaysia. Regarding the scale of enterprises, we distributed the questionnaire to medium- and large-scale information technology companies. In addition, as most variables in this study involved individual self-reports from the sample, questionnaires were limited to employees with at least 1 year of working experience. To assure confidentiality and to reduce participants’ potential concerns about being evaluated, each questionnaire was enclosed within an envelope. This study selected more than 20 information technology companies from Mainland China and Malaysia. Finally, in total, 627 questionnaires from Mainland China and 434 from Malaysia were returned.

In the sample from Mainland China, most respondents are male (72.3%), have a level of education of undergraduate or above (82.7%), are between 30 and 45 years old (80.4%), and have an average of 6.2 working years. In the sample from Malaysia, most respondents are male (73.2%), have a level of education of undergraduate or above (74.6%), are between 30 and 40 years old (63.3%), and have an average of 5.7 working years.

#### Measures

Most of the scales in the questionnaire were adopted from previous studies and modified to suit the research context. The job security scale was developed by Francis and Barling (2005). The scale measures job security and includes five statements, such as “I can keep my current job for as long as I want it” and “This job has retirement security.” To divide market orientation into customer orientation, competitor orientation, and interfunctional coordination, we adopted the scale proposed by Morgan and Vorhies (2018), containing items such as “Student placement shares competitor information.” For self-efficacy, the scale was revised and integrated with six items developed by Rigotti et al. (2008), such as “I can remain calm when facing difficulties in my job because I can rely on my abilities.” Referring to Blue et al. (2001) and Bock and Kim (2002), four items were designed for knowledge sharing intention, for example “I will share my work reports and official documents with members of my organization more frequently in the future.” Besides, referring to de Vries et al. (2006), eight items were developed for knowledge sharing behavior, such as “I like to be informed of what my colleagues know.” All the items were measured using a 5-point Likert scale (1 = totally disagree; 5 = totally agree.

## Results

### Evaluation of the Measurement Model

All latent variables evaluated were found to be reliable in this study, with Cronbach’s α ranging from 0.551 to 0.938. Table 1 shows the reliability of each scale, and the reliabilities in each latent variable have been good, with a Cronbach’s α over 0.70. In order to verify the validity of measurement model, this study conducted confirmatory factor analysis (CFA) *via* PLS-SEM to examine the construct validity, including convergent and discriminant validity. Based on the validity criteria recommended from Hair et al. (2010), CFA results show that standardized factor loadings were higher than 0.5; average variance extracted (AVE) ranges between 0.515 and 0.847; and composite reliability (CR) ranges between 0.769 and 0.949. All three criteria for convergent validity were met, and correlation coefficients were all less than the square root of the AVE within one dimension, suggesting that each dimension in this study had good discriminant validity.

**TABLE 1:** Measurement properties.

		1	2	3	4	5	6	7
1.Self-efficacy		***0.821/00.812***	0.475	0.301	0.240	0.244	0.594	0.546
2.Job security		0.690	***0.771/0.756***	0.282	0.221	0.254	0.422	0.541
3. Customer orientation		0.500	0.541	***0.839/0.826***	0.714	0.630	0.257	0.436
4. Competitor orientation		0.421	0.508	0.775	***0.870/0.860***	0.699	0.276	0.344
5. Interfunction coordination		0.439	0.521	0.716	0.778	***0.852/0.787***	0.280	0.418
6. Knowledge sharing intention		0.756	0.612	0.471	0.410	0.452	***0.869/0.841***	0.460
7. Knowledge sharing behavior		0.684	0.693	0.547	0.493	0.536	0.598	***0.793/0.787***
Mean	Mainland China	3.756	3.580	3.543	3.469	3.406	3.700	3.634
	Malaysia	3.696	3.529	3.151	3.169	3.169	3.478	3.525
SD	Mainland China	0.625	0.567	0.658	0.701	0.704	0.682	0.615
	Malaysia	0.534	0.456	0.638	0.622	0.552	0.594	0.529
α	Mainland China	0.903	0.748	0.915	0.892	0.925	0.891	0.914
	Malaysia	0.896	0.712	0.905	0.881	0.877	0.862	0.910
AVE	Mainland China	0.674	0.595	0.703	0.757	0.726	0.755	0.629
	Malaysia	0.659	0.571	0.683	0.740	0.620	0.708	0.619
CR	Mainland China	0.925	0.854	0.934	0.926	0.941	0.925	0.931
	Malaysia	0.920	0.823	0.928	0.919	0.907	0.906	0.928

*The bold italic diagonal values represent the square root value of AVE; left value belongs to mainland China sample and right value belongs to Malaysian sample. Correlation coefficients below the diagonal belong to mainland China sample and others above the diagonal belong to Malaysian sample.*

### Inner Model Analysis

In this study, the research tools were differentiated, so that rigorous analysis findings could be obtained for the research framework and answers to the research questions to be analyzed could be provided. PLS-SEM is more applicable than Covariance-based structural equation modeling (CB-SEM) under the following conditions: the purpose is to undertake exploratory research for the development of theories; the analysis is conducted for prediction; the structural model is complicated, containing one or more formative constructs; there is a lack of normality for distribution; and latent variable scores are needed for a subsequent analysis (Shiau and Chau, 2016; Hair et al., 2019; Khan et al., 2019). Therefore, in regard to the structural model, the study substituted smart PLS for PLS-SEM to perform the hypothesis verification and comparative analysis.

To assess the structural model, Hair et al. (2017) suggested looking at the *R*^2^, beta (β), and the corresponding *t*-values *via* a bootstrapping procedure with a resample of 5,000. According to claims from Sullivan and Feinn (2012), “while a *p*-value can inform the reader whether an effect exists, it will not reveal the size of the effect. In reporting and interpreting studies, both the substantive significance (effect size) and statistical significance (*p*-value) are essential results to be reported (p. 279).” Before conducting hypotheses testing, this study must ensure that the values of the variance inflation factor (VIF) are less than 5, but the research results showed that the VIF values were between 1 and 1.914. Thus, there were no multicollinearity problems among the latent variables (Hair et al., 2017).

**FIGURE 2:**
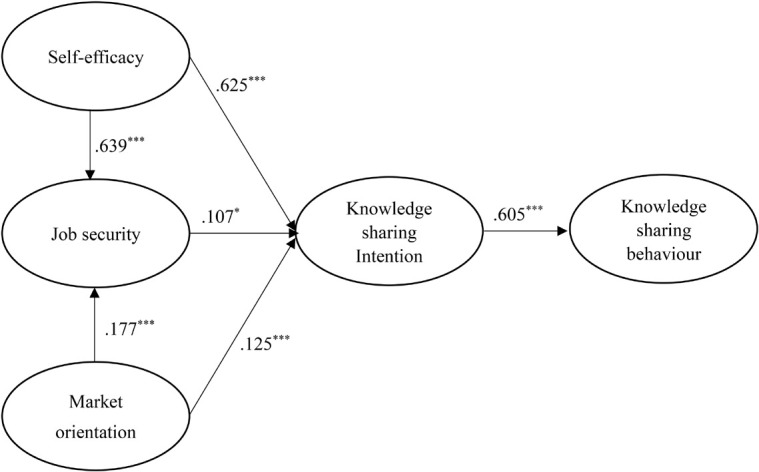
Structural model on Mainland China employees. *p < 0.05; ***p < 0.001.

**FIGURE 3:**
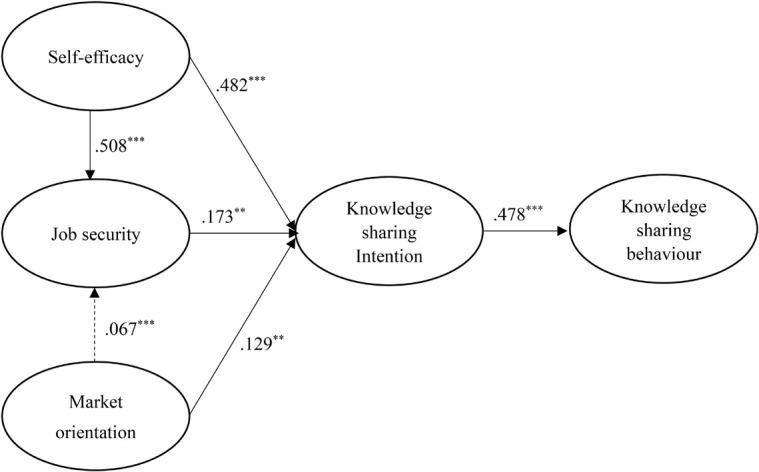
Structural model on Malaysian employees. **p < 0.01; ***p < 0.001.

Figures 2, 3 and Table 2 show the results of the hypothesized relationships and standardized coefficients in Mainland China and Malaysian samples. The results showed that knowledge sharing intention was positively and significantly related to knowledge sharing behavior (β_*Mainland China*_ = 0.605, *f*^2^ = 0.577, *p* < 0.001; β_*Malaysia*_ = 0.478, *f*^2^ = 0.297, *p* < 0.001), supporting H1. Research results showed that job security was positively and significantly related to knowledge sharing intention in both regions (β_*Mainland China*_ = 0.173 *f*^2^ = 0.012, *p* < 0.05; β_*Malaysia*_ = 0.018, *f*^2^ = 0.037, *p* < 0.001), supporting H2.

**TABLE 2:** Results of the hypotheses testing.

Paths	Mainland China		Malaysia		Decision
	β	*p*-value	β	*p*-value	
H1: Knowledge sharing intention→ knowledge Sharing behavior	0.605	0.000	0.478	0.000	Support
H2: Job security→ knowledge sharing intention	0.105	0.018	0.173	0.008	Support
H3: Self-efficacy→ job security	0.639	0.000	0.508	0.000	Support
H4: Self-efficacy→ knowledge sharing behavior	0.625	0.000	0.482	0.000	Support
H5: Market orientation→ Job security	0.177	0.000	0.067	0.102	Partially support
H6: Market orientation→ Knowledge sharing behavior	0.125	0.000	0.129	0.002	Support

The research results showed that self-efficacy was positively and significantly related to job security (β_*Mainland China*_ = 0.639, *f*^2^ = 0.688, *p* < 0.001; β_*Malaysia*_ = 0.508, *f*^2^ = 0.329, *p* < 0.001), supporting H2. Moreover, self-efficacy (β_*Mainland China*_ = 0.625, *f*^2^ = 0.440, *p* < 0.001; β_*Malaysia*_ = 0.482, *f*^2^ = 0.272, *p* < 0.05) was also positively and significantly related to knowledge sharing intention, supporting H3.

In addition, market orientation (β_*Mainland China*_ = 0.177, *f*^2^ = 0.053, *p* < 0.001; β_*Malaysia*_ = 0.006, *f*^2^ = 0.089, *p* > 0.1) was positively and significantly related to job security in Mainland China rather than in Malaysia, partially supporting H4. Similarly, the paths of market orientation → knowledge sharing intention (β_*Mainland China*_ = 0.125, *f*^2^ = 0.028, *p* < 0.001; β_*Malaysia*_ = 0.129, *f*^2^ = 0.026, *p* < 0.01, showed that the relations were positive and significant in Mainland China and Malaysian sample, therefore supporting H5.

### Multiple Group Analysis: Mainland China and Malaysia

Before the cross-cultural comparative analysis, this study first verified whether different background variables lead to differences in Mainland China or Malaysian samples or not. The difference analysis with different background variables all showed significant differences, thereby, MGA was further used to compare path differences. It was confirmed that the measurement pattern was stable. However, in order to avoid overgeneralizing the data-driven patterns and theories, the study followed the suggestion of Hair et al. (2010) to divide the sample data into two groups based on regions (627 Mainland China and 434 Malaysian employees, respectively). Table 3 indicates the structural models’ results and MGA by using nonparametric methods including Henseler’s MGA as recommended by Henseler et al. (2009). Despite the several differences in terms of significant path estimates between the groups, as indicated in Table 3, results showed there are five significant comparison differences between the two groups on all the paths. The results signify that the region plays a moderating role on the relationship among self-efficacy, job security, market orientation, knowledge sharing intention, and knowledge sharing behavior (Hair et al., 2017). The differences in paths comparison among Mainland China vs. Malaysia show that four paths were significant sequentially. These results imply that research framework did differ between the two regions.

**TABLE 3:** Multigroup analysis result.

Paths	β_Mainland China_-β_Malaysia_	*p*-value Henseler’s MGA	Results
H1: Knowledge sharing intention→ knowledge sharing Behavior	0.129	0.017	β_Mainland China_ > β_Malaysia_
H2: Job security→ knowledge sharing intention	–0.068	0.390	–
H3: Self-efficacy→ job security	0.131	0.018	β_Mainland China_ > β_Malaysia_
H4: Self-efficacy→ knowledge sharing Behavior	0.144	0.086	β_Mainland China_ > β_Malaysia_
H5: Market orientation→ job security	0.112	0.047	β_Mainland China_ > β_Malaysia_
H6: Market orientation→ knowledge sharing behavior	–0.004	0.947	–

## Discussion and Conclusion

This study took employees of information technology companies in Mainland China and Malaysian as research samples to test the self-efficacy, job security, market orientation, knowledge sharing intention, and knowledge sharing behavior correlation using the TPB. This study aimed to fill the theoretical gap in the application of Western theories in the Eastern context (Brown et al., 2011; Chang and Edwards, 2015; Lee et al., 2021; Zhao et al., 2021) and increase the generalization of the theory. Based on our research findings, this study aimed to provide the following contributions. First, few studies have verified employees’ knowledge sharing intention and behavior in relation to environmental risk (Thompson et al., 2017). Second, most of the previous studies on the TPB have explored the development process of knowledge sharing conducted by employees within an organization, but only a few studies have made essential contributions concerning employees’ knowledge sharing and global environmental factors. Most of the previous studies on the TPB have explored employees’ process of socialization within the organization, but only a few studies have made essential contributions to the knowledge of individuals’ adoption of general consumer behavior or specific behavior. This study aimed to fill this theoretical gap and enrich the theoretical foundation of the TPB, providing an explanation for and prediction of the organizational behavior of knowledge sharing among organization members. Third, in addition to verifying the research framework in the Asian context, this study included a cross-cultural perspective to ascertain the differences between Mainland China and Malaysia.

The results indicate that the self-efficacy of employees in Mainland China and Malaysia is positively related to their job security and knowledge sharing intention. These results correspond to those of Demir (2015) and Meyers et al. (2019); on the basis of the TPB, they believed that individual factors for employees are conducive to motivating them to devote more attention to their work, improving the confidence required for the work, and enhancing their job security. Our findings are largely consistent with those of these prior studies, supporting the availability of self-efficacy across a range of regions (Hansen et al., 2012). Similarly, the research results show that self-efficacy has a significant and positive effect on knowledge sharing intention in both regions. The research results are similar to those of Le and Lei (2019) and Qi et al. (2019) regarding the TPB in that employees’ sense of cognition commitment helps to decrease absenteeism and increase knowledge sharing. Even in different regions, employees with higher self-efficacy feel a sufficient sense of stability and the availability of resources in a high-risk environment, which facilitates employees’ engagement in knowledge sharing.

Moreover, the results show that market orientation has a more positive and significant effect on job security for employees from Mainland China than for employees from Malaysia. It is also worth noting that employees with a strong market orientation culture actively participate in information collection and application. A strong organizational culture provides employees with feelings of identification with their work and their value for the organization during the collection of market information, thus improving their job security and reducing their job uncertainty. This finding is consistent with the findings of a number of previous studies (Lamm et al., 2015; Kurz et al., 2018; Abbas and Sağsan, 2019) that supported the relationship between market orientation and job security. Most of the studies on market orientation have been conducted on the organizational level to determine the impact on organizational innovation or organizational performance, and few have regarded market orientation as a learning-oriented organizational culture. This study presents an important contribution by verifying that employees can also increase their sense of belonging and value to the organization in the context of cultural influence, thus reducing the staff flow of the organization. Moreover, the relationship between market orientation and knowledge sharing intention is positive and significant. The research findings are consistent with those of previous studies, indicating that organizations with a strong market orientation culture can effectively improve employees’ conscious behavior of knowledge sharing and that this subjective norm can be included in their management practices by means of corporate governance to encourage employees to produce a stronger knowledge sharing intention.

Besides, the findings show that job security is a strong contributor to knowledge sharing intention for employees from Mainland China and Malaysia. These findings are quite consistent with those of Brown et al. (2011) and Kim and Lee (2013), who verified job security cross-sectionally using different samples of employees (Hansen et al., 2012). Moreover, different from the study by Meyers et al. (2019), this study compared two regions in the same model and verified the direct effects of job security generated in the TPB on knowledge sharing. Moreover, the study found that knowledge sharing intention is positively and significantly related to knowledge sharing behavior for employees from both Mainland China and Malaysia. This finding implies that employees carry out effective tacit and explicit knowledge exchange or knowledge sharing within the organization. Through knowledge diffusion and spread, employees can acquire more innovative knowledge and improve their knowledge sharing behavior in different working environments, particularly when facing a tough situation. The positive influence of knowledge sharing intention on knowledge sharing behavior is in line with the findings of previous studies (Kim and Lee, 2013; Kurz et al., 2018; Abbas and Sağsan, 2019), which may improve the TPB’s explanatory utility and cultural relevance to employees who reside in different countries and cultures.

The study also makes a theoretical contribution through its examination of cross-cultural differences’ influence on the relationships among self-efficacy, job security, market orientation, knowledge sharing intention, and knowledge sharing behavior. In this study, through PLS-SEM multigroup analysis, it was explicitly found that the relationship paths between variables differ significantly between employees in Mainland China and employees in Malaysia. In addition to the fact that the path of knowledge sharing intention → knowledge sharing behavior is significantly larger for employees in Mainland China than for employees in Malaysia; employees in Mainland China show a strong positive effect on the remaining paths. The study is consistent with the claims by Lee et al. (2021) and Zhao et al. (2021) that, even within the same geographical location, there are significant differences in the research findings on account of cultural factors, especially in the testing of mediating effects.

### Practical Implications

In sum, our findings suggested some important practical implications for improving the quality of human resources. To establish a relatively complete architecture, in addition to testing the theoretical model of the TPB, this study explored the antecedents influencing employees’ attitude toward knowledge sharing, subjective norms, and perceived behavioral control through decomposition. The findings clarified the structure of the influencing relationship between the variables and the explanatory variations, which provides a reference framework for practitioners to formulate proper strategies, mechanisms, and resource investment in pursuing the performance of knowledge sharing among organization members in knowledge management. When it comes to improving organization members’ positive attitude toward knowledge sharing, an organization should provide appropriate economic benefits (such as pecuniary rewards, promotion, and job security) as external rewards and strengthen the atmosphere of collaboration and mutual benefit to improve the reciprocal relationships of knowledge sharing among organization members. Meanwhile, it should respect the expertise and information owned by each member and inculcate the importance of knowledge sharing for organizational performance.

Furthermore, in regard to improving the perceived behavioral control of organization members, an organization should invest proper resources in establishing software and hardware equipment conducive to knowledge sharing and provide relevant education and training courses for organization members. In the meantime, it should endow them with proper autonomy in work and improve the atmosphere for mutual learning, infrastructure, and knowledge sharing within the organization by establishing a knowledge base and expert directory.

### Research Limitations

The research findings contribute to the literature concerning employees in specific regions and employees’ service innovative behaviors. However, there are still some limitations, which represent subsequent research directions. First, social identity theory has a considerable status in the field of psychology, but only a few studies have taken the relationship between building mechanisms and employees’ knowledge sharing behaviors into consideration. Although this study referred to the TPB and established the building mechanism, and although significant organizational theories can be drawn from the findings, other motivation theories, including the theories of organizational learning (Park and Kim, 2018), self-efficacy (Le et al., 2018), and social exchange (Wu and Lee, 2017), can still be applied to explain how to stimulate innovation behavior among employees in a specific region. Therefore, we suggest conducting subsequent research to apply diversified theoretical models for identifying related psychological dimensions that exert an effect on employees’ knowledge sharing.

Second, employees were required to make self-reports of details with regard to their mental building mechanism as the indicator in the study; this is largely attributed to actual data that are confidential and not easily accessible. Moreover, the theory of planned behavior may contain the interaction pattern between supervisors and employees. In this study, only employees were sampled, and the organizational supporters (supervisors) were not surveyed. In this regard, we suggest that subsequent researchers could add supervisors to the questionnaire to implement cross-level hierarchical regression analysis with the aim of enriching the significance of the theory. Nonetheless, errors may occur in employees’ self-statement of mental conditions. If the actual mental conditions of employees are assessed, the connection between building mechanisms and innovative behaviors may be better understood, considering research ethics. In addition, we propose that subsequent researchers include the contents of interviews and employees’ observations of their work state in their studies to sustain the research findings and draw a comprehensive judgment.

Third, restricted by time and space, a total of 1061 valid copies of questionnaires were sampled. The research objects were classified into employees from Mainland China and employees from Malaysia. Subsequent research can be undertaken both to expand the quantity of samples and the research’s representativeness and to conduct an exploration and comparison of other groups so that extra insights related to organizational behavior management can be offered. Besides, in this study, we did not examine differences between men and women in all the variables. Previous studies have indicated that employees’ gender produces different research outcomes; thus, it is necessary to bring gender into issues related to knowledge sharing intention and behavior. As a consequence, it is suggested that subsequent researchers could add employees’ gender to a comparative analysis to provide more valuable insights.

## Data Availability Statement

The raw data supporting the conclusions of this article will be made available by the authors, without undue reservation.

## Ethics Statement

The studies involving human participants were reviewed and approved by the University of Taipei. The patients/participants provided their written informed consent to participate in this study.

## Author Contributions

MY and PX contributed to the ideas of educational research, collection of data, and empirical analysis. MY contributed to the data analysis, design of research methods, and tables. PX participated in developing a research design, writing, and interpreting the analysis. Both authors contributed to the literature review and conclusions.

## Conflict of Interest

The authors declare that the research was conducted in the absence of any commercial or financial relationships that could be construed as a potential conflict of interest.

## Publisher’s Note

All claims expressed in this article are solely those of the authors and do not necessarily represent those of their affiliated organizations, or those of the publisher, the editors and the reviewers. Any product that may be evaluated in this article, or claim that may be made by its manufacturer, is not guaranteed or endorsed by the publisher.
